# AT-527, a Double Prodrug of a Guanosine Nucleotide Analog, Is a Potent Inhibitor of SARS-CoV-2 *In Vitro* and a Promising Oral Antiviral for Treatment of COVID-19

**DOI:** 10.1128/AAC.02479-20

**Published:** 2021-03-18

**Authors:** Steven S. Good, Jonna Westover, Kie Hoon Jung, Xiao-Jian Zhou, Adel Moussa, Paolo La Colla, Gabriella Collu, Bruno Canard, Jean-Pierre Sommadossi

**Affiliations:** a Atea Pharmaceuticals, Inc., Boston, Massachusetts, USA; b Department of Animal, Dairy & Veterinary Sciences, Utah State University, Logan, Utah, USA; c Università degli Studi di Cagliari, Monserrato, Italy; d Architecture et Fonction des Macromolécules Biologiques, Marseille, France

**Keywords:** AT-527, AT-511, AT-9010, COVID-19, SARS-CoV-2, triphosphate, lung

## Abstract

The impact of severe acute respiratory syndrome coronavirus-2 (SARS-CoV-2), the causative agent of COVID-19, is global and unprecedented. Although remdesivir has recently been approved by the FDA to treat SARS-CoV-2 infection, no oral antiviral is available for outpatient treatment. AT-527, an orally administered double prodrug of a guanosine nucleotide analog, was previously shown to be highly efficacious and well tolerated in hepatitis C virus (HCV)-infected subjects. Here, we report the potent *in vitro* activity of AT-511, the free base of AT-527, against several coronaviruses, including SARS-CoV-2. In normal human airway epithelial cells, the concentration of AT-511 required to inhibit replication of SARS-CoV-2 by 90% (EC_90_) was 0.47 μM, very similar to its EC_90_ against human coronavirus (HCoV)-229E, HCoV-OC43, and SARS-CoV in Huh-7 cells. Little to no cytotoxicity was observed for AT-511 at concentrations up to 100 μM. Substantial levels of the active triphosphate metabolite AT-9010 were formed in normal human bronchial and nasal epithelial cells incubated with 10 μM AT-511 (698 ± 15 and 236 ± 14 μM, respectively), with a half-life of at least 38 h. Results from steady-state pharmacokinetic and tissue distribution studies of nonhuman primates administered oral doses of AT-527, as well as pharmacokinetic data from subjects given daily oral doses of AT-527, predict that twice daily oral doses of 550 mg AT-527 will produce AT-9010 trough concentrations in human lung that exceed the EC_90_ observed for the prodrug against SARS-CoV-2 replication. This suggests that AT-527 may be an effective treatment option for COVID-19.

## Abstract (precis)

The impact of severe acute respiratory syndrome coronavirus-2 (SARS-CoV-2), the causative agent of COVID-19, is global and unprecedented. Although remdesivir has recently been approved by the FDA to treat SARS-CoV-2 infection, no oral antiviral is available for outpatient treatment.

## INTRODUCTION

After multiple severe pneumonia cases were reported in Wuhan, China, in December 2019, a novel coronavirus, severe acute respiratory syndrome coronavirus-2 (SARS-CoV-2), was identified as the causative agent (1). This pathogen causes a potentially life-threatening disease now called coronavirus disease 2019 (COVID-19), which has since rapidly spread around the world, with more than 98 million cases and more than 2.1 million deaths reported globally as of 27 January 2021 according to the World Health Organization (https://www.who.int/emergencies/diseases/novel-coronavirus-2019). The clinical manifestations caused by SARS-CoV-2 infection can range from asymptomatic to fever, cough, dyspnea, myalgia, and headache and to more severe symptoms such as pneumonia, respiratory failure, multiple-organ failure, and eventually death (2). As of January 2021, there are no orally administered therapeutics approved to prevent SARS-CoV-2 infection or to treat COVID-19.

The urgency for treatment options for this public health emergency and the time it takes to develop antiviral therapies have caused interest in repurposing other drugs targeted at other viruses. In fact, remdesivir, the prodrug of an adenosine nucleotide analog originally developed for the treatment of Ebola virus infections, which showed sufficient promise against SARS-CoV-2 (3) and improvement of COVID-19 (4), has recently been approved by the U.S. Food and Drug Administration (FDA) for the treatment of hospitalized patients with COVID-19 (https://www.fda.gov/news-events/press-announcements/fda-approves-first-treatment-covid-19#:∼:text=Today%20C%20the%20U.S.%20Food%20and,of%20COVID%20D19%20requiring%20hospitalization). However, the limited oral bioavailability of remdesivir (5) requires that it be administered via intravenous infusion, thus limiting its use to hospitalized patients. COVID-19 is triggered by an acute viral infection for which antiviral therapeutics will be most effective if given within early stages of the infection when viral load is at its maximum, during rapid replication of SARS-CoV-2 in nasopharyngeal and respiratory epithelium (6). Thus, an orally available direct acting antiviral would be essential for such treatment in an outpatient setting.

AT-527 is an orally available double prodrug of a guanosine nucleotide analog that has demonstrated potent *in vitro* activity against clinical isolates of hepatitis C virus (HCV) and *in vivo* activity in 7-day monotherapy treatment of HCV-infected patients (7) by selectively inhibiting the viral RNA-dependent RNA polymerase (RdRp) (8). Recently, in a phase 2 clinical trial with HCV-infected subjects, AT-527 in combination with daclatasvir, an HCV NS5A inhibitor, was found to be safe and well tolerated at daily oral doses of 550 mg for up to 12 weeks and achieved a high rate of efficacy (9). In the present study, we show that AT-511, the free base of AT-527, has potent antiviral activity when tested *in vitro* against several human coronaviruses, including SARS-CoV-2. The active triphosphate metabolite of AT-527, AT-9010, which cannot penetrate cell membranes and is formed only after intracellular delivery of the prodrug, is produced in substantial amounts in primary human cells of the respiratory tract incubated with AT-511. After oral dosing, the predicted concentration of the active metabolite in pulmonary tissue suggests that AT-527 may be an effective treatment option for individuals infected with COVID-19.

(The *in vitro* virus inhibition data were submitted to an online preprint archive [10].)

## RESULTS

### AT-511 inhibits HCoV-229E.

**FIG 1:**
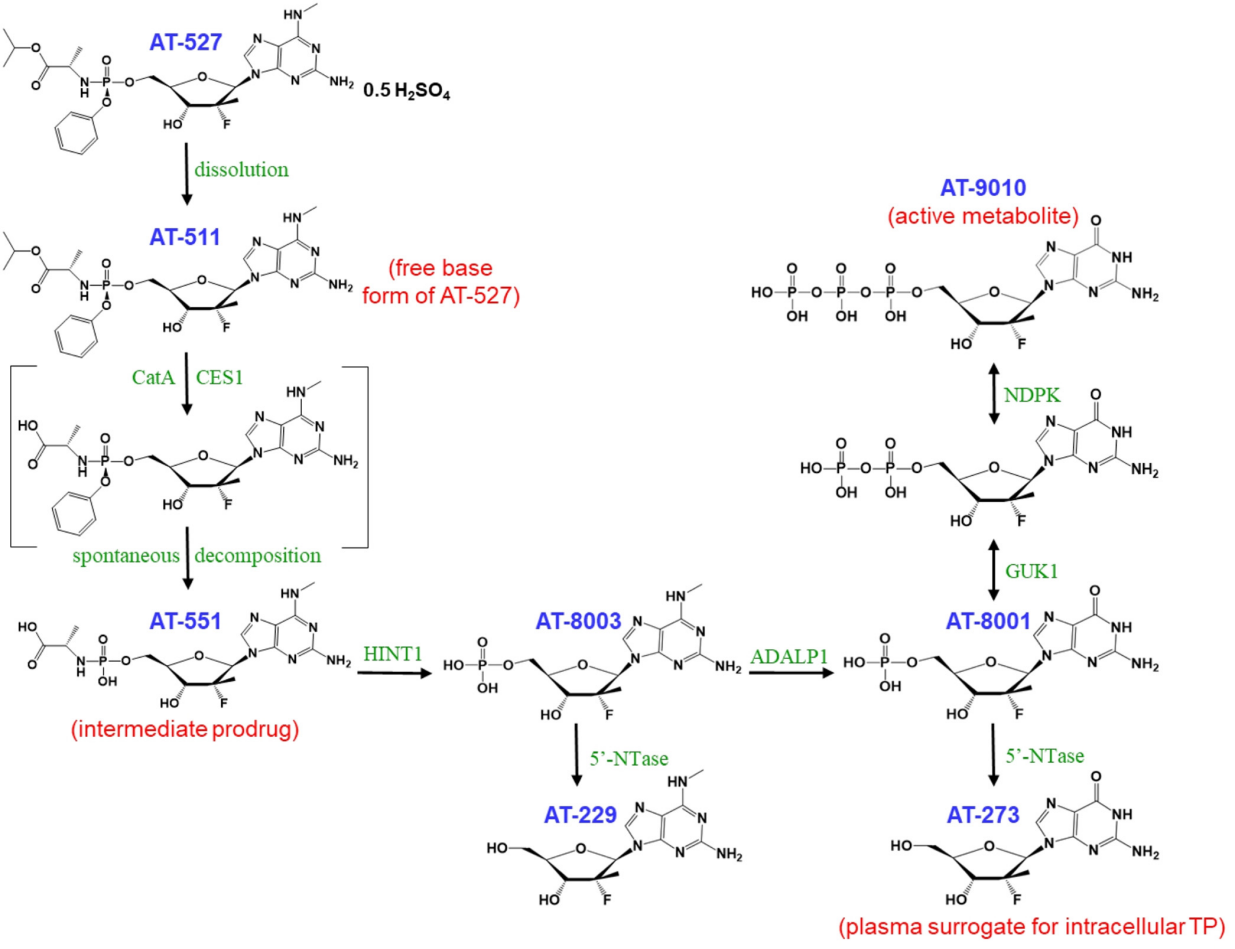
Putative pathway for metabolism of AT-527 to its active triphosphate, AT-9010. Enzymes likely involved in the metabolic pathways include cathepsin A (CatA), carboxylesterase 1 (CES1), histidine nucleotide triad 1 (HINT1), adenosine deaminase-like protein 1 (ADALP1), guanidylate kinase 1 (GUK1), nucleoside diphosphate kinase (NDPK), and 5′-nucleotidase (5′-NTase).

To assess whether AT-511, a 2′-fluoro-2′-methyl guanosine nucleotide prodrug (Fig. 1), had antiviral activity against a seasonal human alpha coronavirus, HCoV-229E, BHK-21 (baby hamster kidney) cells were acutely infected with the virus and exposed to serial dilutions of the drug. After a 3-day incubation, the effective concentration of AT-511 required to achieve 50% inhibition (EC_50_) of the virus-induced cytopathic effect (CPE) from two independent experiments was 1.8 ± 0.3 μM (mean ± standard deviation [SD]) (Table 1). In contrast, the 2′-fluoro-2′-methyl uridine nucleotide prodrug, sofosbuvir, did not inhibit HCoV-229E replication at concentrations as high as 100 μM. Half-maximal cytotoxicity (CC_50_) measured by neutral red staining of compound-treated cells in the absence of virus indicated little to no toxicity was detected from either drug up to 100 μM.

**TABLE 1:** *In vitro* activity of AT-511 and other oral antiviral drugs against various human coronaviruses^*a*^

Virus (genus)	Cell line	Compound	CPE assay		VYR assay EC_90_ (μM [*n*])	Selectivity index (CC_50_/EC_50_)
			EC_50_ (μM [*n*])	CC_50_ (μM)		
HCoV-229E (*Alphacoronavirus*)	BHK-21	AT-511	1.8 ± 0.3 [2]	>100	ND	>55
	BHK-21	Sofosbuvir	>100	>100	ND	ND
	Huh-7	AT-511	1.7 ± 0.1 [2]	>86	1.2 ± 0.1 [2]	>50
HCoV-OC43 (*Betacoronavirus*)	Huh-7	AT-511	ND^*b*^	>86	0.5	>170^*c*^
	RD	AT-511	2.8	>86	2.2	>30
MERS-CoV (*Betacoronavirus*)	Huh-7	AT-511	26 ± 15 [2]	>86	37 ± 28 [2]	>3.3
SARS-CoV (*Betacoronavirus*)	Huh-7	AT-511	ND^*b*^	>86	0.34	>250^*c*^
SARS-CoV-2 (*Betacoronavirus*)	HAE	AT-511	ND^*b*^	>86^*d*^	0.47 ± 0.12 [5]	>160^*c*^
	HAE	Molnupiravir	ND^*b*^	>19^*d*^	2.8 ± 1.0 [3]	>4.9^*c*^

a The activity of AT-511 and other antiviral compounds was measured in cells infected with different coronaviruses, using the cytopathic effect (neutral red dye) assay and/or the virus yield reduction (VYR) assay as described in Materials and Methods, to determine the effective concentration required to achieve 50% inhibition (EC_50_) of the virus-induced cytopathic effect (CPE), the concentration to reduce virus yield by 1 log_10_ (EC_90_), and the cytotoxic concentration of the drug to cause death to 50% of viable cells without virus (CC_50_). Values represent results from single or multiple (mean ± SD [n]) experiments.

b Not determined because no cytopathic effect was produced by this virus in this cell line.

c CC_50_/EC_90_, since EC_50_ could not be determined by measuring CPE in the neutral red assay.

d Cytotoxicity assessed by visual inspection of cell monolayers.

### AT-511 inhibits replication of several human coronaviruses.

In addition to HCoV-229E, we assessed the *in vitro* potency of AT-511 against HCoV-OC43 (another seasonal human coronavirus strain) infection and against MERS-CoV and SARS-CoV (the coronavirus strains responsible for Middle East respiratory syndrome and severe acute respiratory syndrome, respectively) infection. We used Huh-7 (human hepatocellular carcinoma) cells, which have been shown to efficiently form the active triphosphate (TP) metabolite of AT-511 (8) and RD (human rhabdomyosarcoma) cells, whose ability to form the active metabolite is unknown. After a 5-day (HCoV-229E and -OC43) or 7-day (MERS- and SARS-CoV) incubation with virus, an EC_50_ value of 1.7 μM for HCoV-229E confirmed our findings in BHK-21 cells. Since a CPE was only observed in Huh-7 cells infected with HCoV-229E or MERS-CoV, a virus yield reduction (VYR) assay was used to measure the effective concentration required to reduce infectious virus in the culture medium by 90% (EC_90_) in all assays except for the one conducted in BHK-21 cells. In Huh-7 cells infected with HCoV-229E, HCoV-OC43, and SARS-CoV, EC_90_ values ranged from 0.34 to 1.2 μM, whereas the EC_90_ against MERS-CoV was 37 ± 28 μM (mean ± SD) (Table 1). Cytotoxicity due to AT-511 was minimal to absent up to 86 μM, the highest concentration tested.

### AT-511 inhibits SARS-CoV-2 replication in a human airway epithelial tissue model.

Since a robust *in vitro* antiviral assay using Huh-7 or RD cells did not appear to be readily developable without using an unusually high multiplicity of infection (MOI), and a more relevant model using primary human airway epithelial (HAE) cells had already been developed, we turned to this system to assess the antiviral effect of AT-511 against SARS-CoV-2. HAE cells from a single healthy nonsmoking donor were incubated with SARS-CoV-2 in the presence of 0 to 86 μM (0 to 50 μg/ml) AT-511 for 2 h. Virus was then removed, and after a 5-day incubation with continued exposure to drug, virus yield was quantitated. The average EC_90_ value from five separate experiments was 0.47 ± 0.12 μM (mean ± SD) (Table 1). The CC_50_ value of >86 μM (Table 1) reflects the lack of any observed toxicity at the highest concentration tested. These measurements were in the same range as those obtained for HCoV-OC43 and SARS-CoV. For comparison, the mean EC_90_ value in these HAE cell preparations for molnupiravir, the 5′-isobutyl ester oral prodrug of the nucleoside analog N^4^-hydroxycytidine, reported to have *in vitro* and *in vivo* activity against SARS-CoV-2 (11), was 2.8 ± 1.0 μM (Table 1). Intra-assay evaluations demonstrated that molnupiravir was 5 to 8 times less potent than AT-511.

### AT-9010, the active TP metabolite of AT-527, is formed in primary human cells of the respiratory tract.

**FIG 2:**
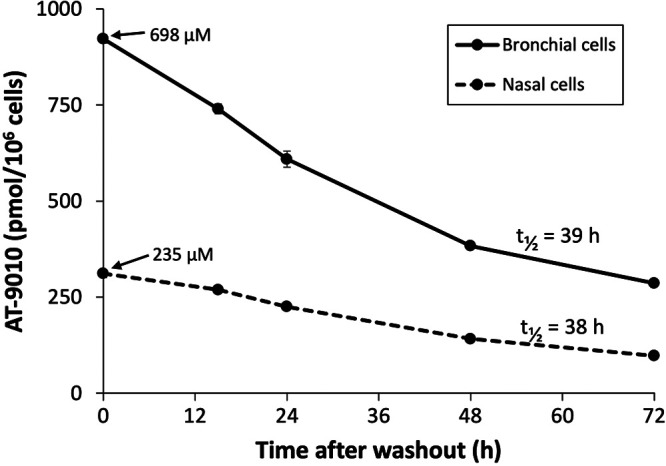
Formation and intracellular half-life of AT-9010 in primary human bronchial and nasal epithelial cells. After cells were exposed to 10 μM AT-511 for 8 h, they were rinsed, and fresh medium was applied for 0, 15, 24, 48, and 72 h post-drug removal. Cells were collected and extracted at each time point (*n* = 3), the active TP was measured by LC-MS/MS, and the half-life (*t*_1/2_) was determined as described in Materials and Methods. Data are expressed at means ± SEs.

AT-527 requires metabolism by the host cell to form the pharmacologically active TP, AT-9010, to inhibit virus replication (Fig. 1). We first tested MRC-5 (human lung fibroblast) cells, which are typically susceptible to infection by various viruses; however, minimal formation of the active TP was observed in triplicate 8-h incubations with 10 μM AT-511 (26.5 ± 0.5 pmol/10^6^ cells), in contrast to the efficient production of AT-9010 in parallel incubations with Huh-7 cells (182 ± 6 pmol/10^6^ cells). When primary human bronchial and nasal epithelial cells from single healthy donors were incubated in triplicate with 10 μM AT-511 for 8 h, these cells readily formed the active TP with a half-life (*t*_1/2_) of 38 to 39 h (Fig. 2). Using the calculated average volume of 1,320 μm^3^ per alveolar type I and II epithelial cells (12), mean intracellular AT-9010 concentrations rose to 698 ± 15 μM in human bronchial epithelial (HBE) cells and 235 ± 14 μM in human nasal epithelial (HNE) cells. Little or no cytotoxicity was observed in parallel incubations with 100 μM AT-511, with HBE and HNE cell viabilities of 96.8% ± 1.4% and 97.3% ± 2.3% relative to their corresponding vehicle controls of 100% ± 1.0% and 100% ± 3.1%.

### AT-9010 is formed in tissues of nonhuman primates after oral dosing of AT-527.

**FIG 3:**
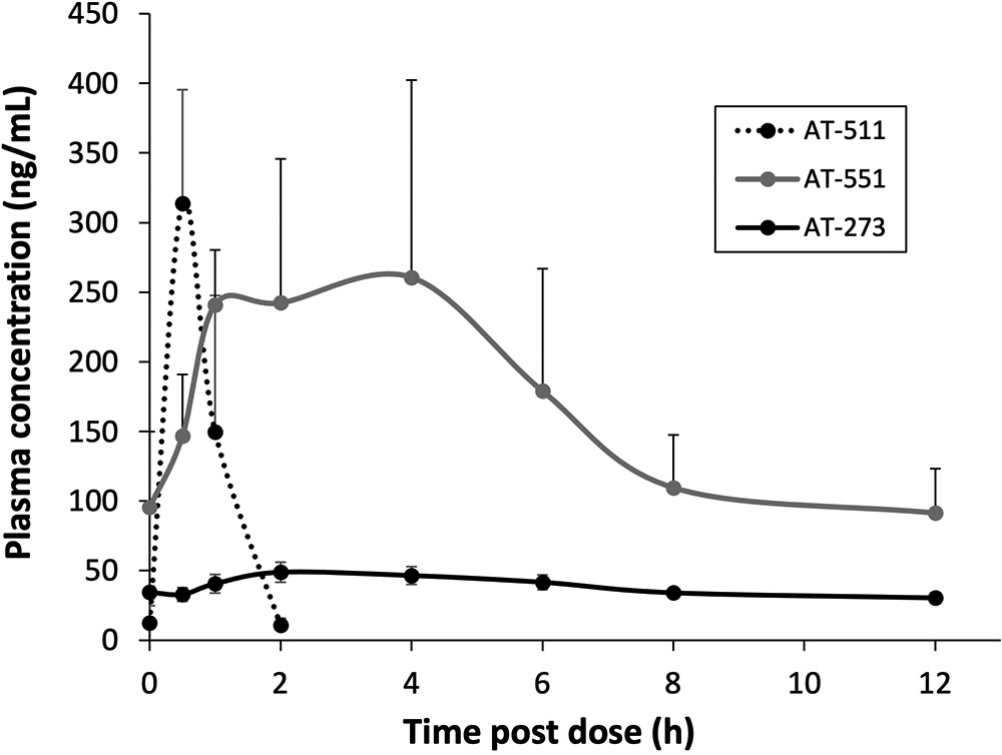
Plasma profiles of AT-511 and its metabolites in nonhuman primates after oral doses of AT-527. Cynomolgus monkeys (*n* = 3) were orally administered a loading dose of 60 mg/kg AT-527 followed by 30 mg/kg maintenance doses every 12 h for 3 days to achieve steady state. Blood was collected preceding and at 0.5, 1, 2, 4, 6, 8, and 12 h after the 5th dose, and plasma AT-511, AT-551, and AT-273 concentrations were determined by LC-MS/MS. Data are expressed as means ± SEs.

**FIG 4:**
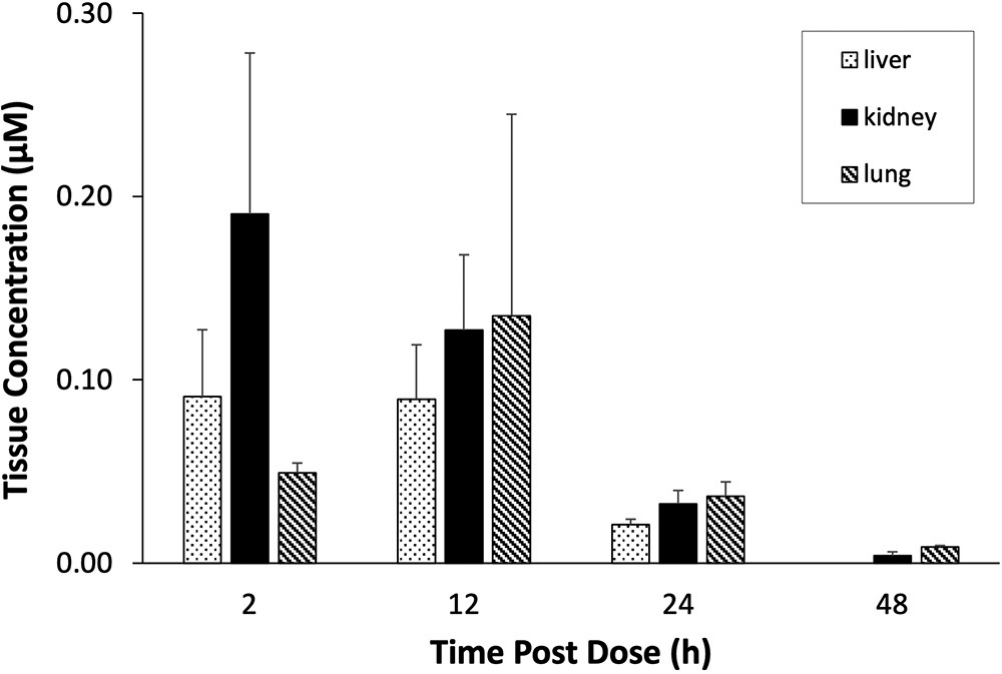
Tissue concentrations of AT-9010 in nonhuman primates. Cynomolgus monkeys were given a loading dose of 60 mg/kg AT-527 followed by 30 mg/kg maintenance doses every 12 h for 3 days to achieve steady state. Tissue samples were collected under anesthesia at the indicated time points after the last dose, flash frozen, and homogenized, and AT-9010 concentrations were determined by LC-MS/MS. Data are expressed as means ± SEs (*n* = 3).

The steady-state pharmacokinetics (PK) of AT-511 and its metabolites in nonhuman primates (NHPs) after twice daily (BID) oral administration of AT-527 for 5 days at 30 mg/kg body weight, a dose allometrically scaled from the proposed clinical dose of 550 mg BID, are reported in Table 2. In this study, the PK values of the parent prodrug AT-511 and its plasma metabolites were comparable to previously reported results (8). AT-511 was rapidly converted to the intermediate prodrug AT-551 and to AT-273, the surrogate plasma marker for intracellular concentrations of AT-9010 (Fig. 3). The AT-9010 concentration of 0.14 ± 0.11 μM (mean ± standard error [SE], *n* = 3) in NHP lung tissue at 12 h after the last dose (steady-state trough level) (Fig. 4) closely reflected the concurrent plasma AT-273 concentration of 0.12 ± 0.02 μM (Fig. 3). The AT-9010 concentration in NHP kidney at 12-h postdose (0.13 ± 0.04 μM) (Fig. 4) was similar to that in lung, but the level of the TP in NHP liver tissue was 1.6 times lower than in lung, at 0.09 ± 0.03 μM (Fig. 4). These concentrations were calculated using noninterstitial volumes of 0.75 and 0.9 ml/g lung and liver, respectively, and an assumed volume of 0.83 ml/g kidney (13). In contrast to that for lung and kidney, for which 48-h concentrations could be reasonably estimated by extrapolation below the assay calibration curves for all but one of the three kidney samples, no peak corresponding to AT-9010 was detected in any of the liver samples at the 48-h postdose time point. The estimated half-lives of the active TP in lung and kidney tissues were 9.4 and 8.5 h, respectively. For liver tissue, the *t*_1/2_ of AT-9010 was apparently shorter but could not be estimated from the results obtained. Using data obtained from a separate study at earlier time points (4 and 8 h) where AT-9010 concentrations were measurable in NHP liver after similar repeated oral dosing of AT-527 (S. Good, unpublished data) combined with the liver concentrations obtained at the 12- and 24-h time points from the present study, the *t*_1/2_ of AT-9010 in NHP liver tissue was estimated to be approximately 4 h.

**TABLE 2:** Plasma pharmacokinetic parameters in NHPs after oral administration of AT-527^*a*^

Compound	*C*_max_^*b*^ (μM)	*C*_12_^*c*^ (μM)	*T*_max_^*d*^ (h)	*t*_1/2_ (h)	AUC_0–12_^*e*^ (μM·h)
AT-511 (parent prodrug)	0.64 ± 0.08	ND	0.5–1	0.7	0.44 ± 0.09
AT-551 (intermediate prodrug)	0.68 ± 0.25	0.20 ± 0.07	1–4	8.8	4.35 ± 1.77
AT-273 (plasma surrogate for intracellular TP)	0.16 ± 0.02	0.10 ± 0.01	2	16	1.56 ± 0.18

a Nonhuman primates (NHPs) were given a loading dose of 60 mg/kg AT-527, followed by five doses of 30 mg/kg every 12 h. Plasma samples were collected prior to the 5th dose and then 0.5, 1, 2, 4, 6, 8, 12 (preceding the 6th dose), and 14 h thereafter and analyzed for AT-511 and its metabolites by LC-MS/MS. Data are expressed as means ± SEs (*n* = 3). ND, not detected. The analytes in the putative metabolic pathway for AT-527 are shown in Fig. 1.

b *C*_max_, maximum concentration across the time points measured.

c *C*_12_, concentration at 12 h.

d *T*_max_, time at which *C*_max_ was observed.

e AUC_0–12_, area under the plasma concentration versus time curve, from 0 to 12 h.

### Human hepatocytes form more AT-9010 than primary NHP hepatocytes.

**FIG 5:**
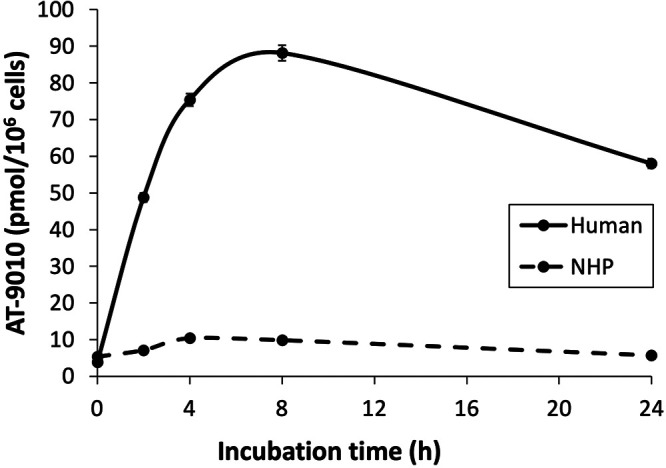
AT-9010 concentrations in primary human and cynomolgus monkey hepatocytes. Hepatocytes were incubated with 10 μM AT-511 for 0, 2, 4, 8, and 24 h in triplicate. They were then washed, extracted, and analyzed for AT-9010 by LC-MS/MS. Data were used from reference 8 and are expressed as means ± SEs.

To assess whether NHPs metabolize AT-527 to its active TP similarly to humans, primary hepatocytes from the two species were incubated with 10 μM AT-511 for 24 h (8). Based on a comparison of area under the concentration-time curve from 0 to 24 h (AUC_0–24_) values, AT-9010 concentrations were up to 7 times greater in human than in NHP hepatocytes (Fig. 5). Using a hepatocyte volume of 3.4 × 10^−9 ^ml (14) to calculate the intracellular concentration, AT-9010 peaked at 8 h in human hepatocytes at 26 ± 1 μM, while the highest concentration in NHP hepatocytes was 3.1 ± 0.1 μM at 4 h.

## DISCUSSION

From our previous studies with AT-527 for the treatment of hepatitis C virus infections, we had observed that Huh-7 cells efficiently phosphorylated AT-511 to the active TP (8), and so we tested the antiviral activity of AT-511 against several coronaviruses using this cell line. While active against HCoV-229E, HCoV-OC43, and SARS-CoV, AT-511 had minimal activity against MERS-CoV (Table 1). This result was initially puzzling, since nucleotide and nucleotide analogue selection is achieved at the highly conserved amino acid motifs A and C involved in phosphodiester bond formation and at motifs F and B that participate in nucleotide channeling and binding at the active site of the coronavirus RdRp, which resides in the nonstructural protein (nsp) 12 gene product and is activated by its processivity cofactors nsp7 and nsp8 (15). Additionally, with the similar ribose modifications in both AT-511 and sofosbuvir, it is unlikely that their differential activity would be due to selective excision by the coronavirus exonuclease carried by nsp14 (16). It should be noted, however, that the nsp12 of SARS-CoV-2 and other coronaviruses contains two functional domains, including RdRp and nidovirus RdRp-associated nucleotidyltransferase (NiRAN), which serves to prime the RdRp for RNA synthesis (17, 18). Recent data indicate that AT-9010 is a potent inhibitor of NiRAN (our unpublished data), the function of which is essential for viral replication (19). Thus, our results suggest that, in addition to acting as a nonobligate chain terminator of viral RNA replication, the TP formed from AT-511 may target the nucleotide binding site in the NiRAN domain of nsp12 whose inhibition would account for the antiviral effect. If such is the case, the differential sensitivity pattern to AT-511 suggests that the NiRAN domain in MERS-CoV differs from that in the other strains tested.

In contrast to a recent publication (3), an assay to assess the activity of drug candidates against SARS-CoV-2 replication in Huh-7 cells was not readily attainable in our hands. Moreover, although MRC-5 cells are able to be infected by SARS-CoV-2 (20), this cell line phosphorylated AT-511 poorly, which is consistent with the lack of activity we observed when AT-511 was tested against HCoV-229E in these cells (8). This may also be true for Vero cells, an African green monkey kidney cell line often used to study viral infection and vaccines, in which AT-511 was previously shown to be inactive or weakly active against other viruses (8); so, we turned to HAE cells, a highly relevant *in vitro* model of human respiratory tract tissue which has been established as a more representative system than cell lines for studying SARS-CoV-2 infection (21). These primary cells form polarized monolayers, the ciliated apical side of which is exposed to air and produces a mucin layer, consistent with the physiology of human airways (11), which highlights the importance of the finding that AT-511 is metabolized to its active TP in a cell system relevant to COVID-19.

The PK parameters in NHPs after oral administration of AT-527 in the present study were similar to data reported in previous studies (8). It was reassuring to observe that the 12-h concentration of AT-273 in plasma, the surrogate marker for intracellular AT-9010, was similar in the lung and kidney tissues of these animals. Moreover, more of the active TP was found in lung than in liver, for a longer period of time. However, NHPs may not be the best model to assess human response to AT-527, since the *in vitro* formation of AT-9010 in primary hepatocytes was 7-fold lower in NHPs than in humans.

**FIG 6:**
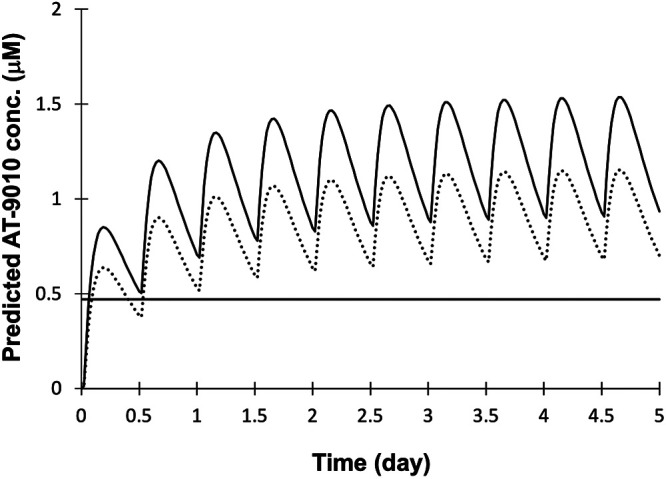
Simulated AT-9010 concentrations in human lung. Human lung AT-9010 concentrations were predicted for 550-mg BID dosing using published data from subjects given daily 550-mg doses of AT-527 as described in Materials and Methods. The solid curve represents predicted lung concentrations of the active TP metabolite after correcting for the AT-9010 lung-to-liver concentration ratio of 1.6 observed in cynomolgus monkeys at 12 h after the last of 6 BID doses of AT-527 as described for Fig. 4. The dotted curve represents predicted lung concentrations of the active TP metabolite after correcting for the AT-9010 lung-to-AT-273 plasma ratio of 1.2 observed in the same monkeys at the same time point. The horizontal line represents the EC_90_ of AT-511 against SARS-CoV-2 in HAE cells *in vitro* (0.47 μM).

Since plasma levels of the nucleoside metabolite AT-273 arise from catabolism of its intracellular phosphorylated forms and the TP is the predominant form in all cells and tissues examined to date (8), plasma levels of AT-273 are considered a surrogate for intracellular levels of AT-9010. This hypothesis is supported by the observation that plasma AT-273 concentrations accurately reflect the antiviral activity of escalating doses of AT-527 in HCV-infected subjects (7). However, some tissues may contribute to this pool more than others. The findings that the 12-h steady-state AT-9010 levels in NHP lung were 1.6-fold higher than those in liver and that the *in vitro t*_1/2_ of the TP in HBE and HNE cells was 4-fold longer than that in NHP and human hepatocytes (both 10 h) suggest that human liver levels of AT-9010 may be lower than those in lung and that predictions of the latter based on circulating plasma levels of AT-273 may be underestimated. Nevertheless, the kinetics of human lung AT-9010 levels were simulated for a 550-mg BID dose regimen for 5 days (Fig. 6) using published plasma AT-273 data from subjects given daily 550-mg doses of AT-527 (7) amplified by a factor of 1.6 based on the assumption that the observed lung-to-liver AT-9010 concentration ratio in NHPs is applicable to humans as well. The resulting predicted steady-state peak and trough levels for the active TP in human lung for this dose regimen are 1.5 and 0.9 μM, respectively. A second approach to predicting human lung AT-9010 concentrations used the same simulated plasma AT-273 data but corrected the plasma values by a factor of 1.2, which is the ratio of the mean steady-state 12-h lung AT-9010 concentration in NHPs to that of AT-273 in plasma. This prediction provided respective estimates of 1.1 and 0.7 μM for peak and trough human lung TP concentrations. According to either method, the predicted human lung levels of AT-9010 exceed the EC_90_ value observed against SARS-CoV-2 replication in HAE cells from within a few hours after the first dose through the end of the dosing period. By these calculations, AT-527 is an attractive treatment option for patients with COVID-19.

To date, human safety and tolerability have been confirmed in more than 40 HCV-infected patients treated orally with once-daily administration of 550 mg of AT-527 in combination with daclatasvir for 7 days and up to 12 weeks (9). As we have reported here using two independent methods, excellent pharmacokinetics (estimated oral bioavailability of at least 50% and a long intracellular *t*_1/2_ of the active metabolite) associated with a dosing regimen of 550 mg BID should provide lung exposures to the active TP metabolite consistently above the drug’s *in vitro* EC_90_ against SARS-CoV-2 replication and, therefore, may lead to an effective antiviral treatment. A phase 2 clinical trial (ClinicalTrials registration no. NCT04396106) is currently ongoing to evaluate the safety and efficacy of AT-527 in COVID-19 patients.

## MATERIALS AND METHODS

### Cell lines, viruses, and test compounds.

BHK-21 (baby hamster kidney; American Type Culture Collection [ATCC], Manassas, VA) cells were maintained in minimum essential medium with Earle’s salts (MEM-E) containing 1 mM sodium pyruvate and 25 μg/ml kanamycin, supplemented with 10% fetal bovine serum (FBS). RD (human rhabdomyosarcoma) cells, MRC-5 (human lung fibroblast) cells, and the Vero 76 cells used for the virus yield reduction assay were also obtained from ATCC and maintained similarly using Eagle’s minimum essential medium (EMEM), FBS, and antibiotics. Huh-7 (human liver carcinoma; AcceGen Biotechnology, Fairfield, NJ) cells were maintained in Dulbecco’s modified Eagle medium (DMEM) supplemented with 10% FBS, 100 μg/ml penicillin, and 100 μg/ml streptomycin (Lonza, Walkersville, MD). The differentiated normal human airway epithelial (HAE) cells (EpiAirway AIR-100 or AIR-112) were prepared by MatTek Corporation (Ashland, MA), and their culture and infection are described below. Human bronchial and nasal epithelial cells from single healthy nonsmoker donors were obtained from PromoCell GmbH (Heidelberg, Germany) and maintained in their proprietary airway epithelial cell growth medium with penicillin and streptomycin added. All cell cultures were maintained at 37°C in an atmosphere of 5% CO_2_ and ≥95% humidity. Infections were performed in EMEM supplemented with 5% FBS and 50 μg/ml gentamicin.

The seasonal human coronaviruses (HCoV-229E and HCoV-OC43) were obtained from ATCC (Manassas, VA). MERS-CoV (EMC), SARS-CoV (Urbani), and SARS-CoV-2 (USA-WA1/2020) were supplied by the Centers for Disease Control and Prevention, Atlanta, GA. Sofosbuvir and molnupiravir were purchased from Pharma Sys, Inc. (Cary, NC) and MedChemExpress (Monmouth Junction, NJ), respectively. AT-527, its free base AT-511, and its metabolites AT-551 and AT-273 were prepared for Atea Pharmaceuticals by Topharman Shanghai Co., Ltd., Shanghai, China. AT-9010 and the TP internal standards used to quantify AT-9010 were synthesized by NuBlocks (Oceanside, CA). Stock solutions were prepared in dimethyl sulfoxide (DMSO) and stored at −20°C. The virus studies were conducted at Utah State University (Logan, UT). The other cell studies were carried out at WuXi AppTec Laboratory Testing Division (Cranbury, NJ).

### HAE cell preparations.

HAE cells were grown on 6-mm mesh disks and arrived in kits with either 12- or 24-well transwell inserts. During transportation, the model tissues were stabilized on a sheet of agarose, which was removed upon receipt. One insert was estimated to consist of approximately 1.2 × 10^6^ cells. Kits of cell inserts (EpiAirway AIR-100 or AIR-112) originated from a single donor, number 9831, a 23-year-old, healthy, nonsmoking Caucasian male. The cells form polarized monolayers, the apical side of which is exposed to air and creates a mucin layer. Upon arrival, the cell transwell inserts were immediately transferred to individual wells of a 6-well plate according to the manufacturer’s instructions, and 1 ml of MatTek’s proprietary culture medium (AIR-100-MM) was added to the basolateral side, whereas the apical side was exposed to a humidified 5% CO_2_ environment. Cells were given a 24-h equilibration period before the start of the experiment, at which time, the mucin layer, secreted from the apical side of the cells, was removed by washing 3 times with 400 μl prewarmed 30 mM HEPES-buffered saline solution. Culture medium was replenished following the wash steps.

### HCoV-229E infection and treatment of BHK-21 cells.

AT-511 and sofosbuvir were dissolved in DMSO at 100 mM and then diluted in growth medium to final concentrations of 100, 20, 4, and 0.8 μM (two 24-well replicate plates each). After BHK-21 cells were grown to confluence in 96-well plates, growth medium was replaced with fresh maintenance medium (growth medium with 1% inactivated FBS in place of 10% FBS) containing serially diluted test compound and HCoV-229E at a multiplicity of infection (MOI) of 0.01. Uninfected cells in the presence of serially diluted compound were used to assess the cytotoxicity of compounds. After a 3-day incubation at 37°C in a humidified 5% CO_2_ atmosphere, cell viability was determined by the 3-(4,5-dimethyl-2-thiazolyl)-2,5-diphenyl-2H-tetrazolium bromide (MTT) method (22). The effective concentrations of test compound required to prevent virus-induced cytopathic effect (CPE) by 50% (EC_50_) and to cause 50% cell death in the absence of virus (CC_50_) were calculated by regression analysis.

### Coronavirus infection and treatment of Huh-7 and RD cells.

The antiviral activity of AT-511 was evaluated against human *Alphacoronavirus* (229E) and *Betacoronavirus* (OC43), MERS (EMC), and SARS (Urbani) using a neutral red assay to determine inhibition of virus-induced and compound-induced CPE and using a virus yield reduction (VYR) assay as a second independent determination of the inhibition of viral replication.

### Neutral red assay.

Test compounds were dissolved in DMSO at a concentration of 10 mg/ml and serially diluted using eight half-log dilutions so that the highest test concentration was 50 μg/ml (86 μM). Each dilution was added to 5 wells of a 96-well plate with 80 to 100% confluent Huh-7 or RD cells (OC43 only). Three wells of each dilution were infected with virus, and two wells remained uninfected as toxicity controls. Six untreated wells were infected as virus controls, and six untreated wells were left uninfected to use as virus controls. Viruses were diluted to achieve MOIs of 0.003, 0.002, 0.001, and 0.03 CCID_50_ (50% cell culture infectious dose) per cell for 229E, OC43, MERS, and SARS coronaviruses, respectively. Plates were incubated at 37°C in a humidified atmosphere containing 5% CO_2_. On day 5 (HCoV-229E and -OC43) or day 7 (MERS- and SARS-CoV) postinfection, when untreated virus control wells reached maximum CPE, the plates were stained with neutral red dye for approximately 2 h (±15 min). Supernatant dye was removed, wells were rinsed with phosphate-buffered saline (PBS), and the incorporated dye was extracted in 50:50 Sorensen citrate buffer-ethanol for >30 min. The optical density was read on a spectrophotometer at 540 nm and converted to percentage of controls. The concentrations of test compound required to prevent virus-induced CPE by 50% (EC_50_) and to cause 50% cell death in the absence of virus (CC_50_) were calculated (23, 24). The selective index is the CC_50_ divided by EC_50_ except where indicated.

### Virus yield reduction assay.

As previously published (25), Vero 76 cells were seeded in 96-well plates and grown overnight (37°C) to confluence. A sample of the supernatant fluid from each compound concentration or vehicle control incubated with virus and cells as described above for the neutral red assay was collected on day 3 postinfection (3 wells pooled) and tested for virus titer using a standard endpoint dilution CCID_50_ assay and titer calculations using the Reed-Muench equation (26). The concentration of compound required to reduce virus yield by 90% (EC_90_) was determined by regression analysis.

### SARS-CoV-2 infection and treatment of HAE cells.

SARS-CoV-2 virus was diluted in AIR-100-MM medium before infection to yield an MOI when added to cultures of approximately 0.0015 CCID_50_ per cell. Using four or six 5-fold or 1-log serial dilutions, AT-511 or molnupiravir (final top concentration 5 μg/ml; 8.6 or 19 μM, respectively) was applied to the cells (120 μl to the apical side, and 1 ml to the basal side), while virus (120 μl) was applied only to the apical side. As a virus control, some of the cells were treated with cell culture medium only. After a 2-h infection incubation, the apical medium was removed, and the basal medium was replaced with fresh compound or medium (1 ml). The cells were maintained at the air-liquid interface. On day 5, cytotoxicity (CC_50_ values) in the uninfected compound-treated inserts was estimated by visual inspection, and the basal medium was removed from all inserts and discarded. Virus released into the apical compartment of the HAE cells was harvested by the addition of 400 μl of culture medium that was prewarmed at 37°C. The contents were incubated for 30 min, mixed well, collected, thoroughly vortexed, and plated on Vero 76 cells for VYR titration. Separate wells were used for virus control, and duplicate wells were used for untreated cell controls. Virus titers from each treated culture were determined as described above. This experiment was repeated using 1 and 50 μg/ml (1.7 and 86 μM) as the top concentration of AT-511.

### Formation of AT-9010 *in vitro*.

Human bronchial and nasal epithelial cells were cultured, harvested with DetachKit 2 (PromoCell GmbH), and plated according to the vendor’s instructions at 1 × 10^6^ cells per well. Once the cells were confluent, 10 μM or 100 μM AT-511 was added for 8 h of incubation. Cell viability and density, which were measured using an automatic cell counter (Cellometer K2, Nexcelom) after staining cells with acridine orange and propidium iodide, were determined in a subset of samples before plating, at confluence before the addition of drug, and at the end of the incubation period along with untreated cells used as vehicle controls. The treated cells were given a 72-h washout. That is, the medium was removed, and the cell layer was rinsed with HEPES-buffered saline solution, followed by the addition of fresh cell culture medium without drug. At 0, 15, 24, 48, and 72 h postwashout, the medium was removed, the cells were rinsed with the HEPES solution and collected, and samples were extracted in ice-cold 60% methanol (MeOH) as described above and analyzed for concentrations of AT-511 and AT-9010 by liquid chromatography-tandem mass spectrometry (LC-MS/MS). The CellTiterGlo assay kit was also used to confirm cell viability at the end of the 8-h incubation. The half-life (*t*_1/2_) of the triphosphate was determined according to the following equation: *t*_1//2_ = ln 2/*k*, where *k* is the elimination rate constant determined by fitting the percent remaining as a function of incubation time to a one-phase exponential decay nonlinear regression model using GraphPad Prism. Data points at a *t* value of 0 were excluded from the calculations.

### Animal welfare.

The animal study described herein was conducted at WuXi AppTec (Suzhou, China) in strict compliance with AAALAC International and NIH guidelines as described in the Guide for the Care and Use of Laboratory Animals, the National Research Council–ILAR, revised 2011, and the People’s Republic of China, Ministry of Science and Technology Regulations for the Administration of Affairs Concerning Experimental Animals (2017). The study was conducted in full compliance with protocols that were reviewed and approved by WuXi AppTec’s Institutional Animal Care and Use Committee (IACUC) prior to study initiation (IACUC number SZ20200529), and all animals were assessed as to their general health by a member of the WuXi AppTec veterinary staff upon arrival and prior to being placed in the study. All animals were housed in rooms with controlled temperature (18 to 26°C), relative humidity (40% to 70%), and light cycle (12 h artificial light and 12 h dark), with 10 to 20 air changes/h, and were provided with manipulatives/enrichment toys.

### Nonhuman primate PK study.

Male nonnaive cynomolgus monkeys, at least 2 years old and 2 kg in body weight, were obtained from Hainan Jingang Laboratory Animal Co., Ltd. (Hainan, China), group housed during the 5-day acclimation period or individually housed during the study in stainless steel mesh cages, provided *ad libitum* access to reverse osmosis (RO) water, fed twice daily at approximately 12 PM (2 h postdosing) and 5 PM (5-h interval) with ∼60 g certified monkey diet each feed (Beijing Vital Keao Feed Co., Ltd., Beijing, China), and given daily treats of fresh fruit. Twelve monkeys were orally dosed twice a day (BID) with AT-527 for 3 days to achieve steady-state levels, and then plasma and tissue samples were collected to determine pharmacokinetics. Specifically, animals were given a 60-mg/kg loading dose, followed by five 30-mg/kg doses every 12 h. This regimen was determined from allometrically scaling the human clinical dosing of 1,100-mg loading dose, followed by 550 mg BID. AT-527 was uniformly suspended in water by stirring at room temperature for 30 min before being administered to the animals by gavage tube, followed by a vehicle flush of 3 ml (approximately 3 times the gavage tube volume). Blood samples (∼0.5 ml) were collected from three animals prior to the 5th dose, at 0.5, 1, 2, 4, 6, 8, and 12 h (the first and last time points preceding the 5th and 6th doses), and at sacrifice (2 h after the last dose). Blood samples were collected from the rest of the animals, in groups of three, at 12, 24, and 48 h after the last dose, before they were sacrificed for tissues. Animals were observed for any unusual or adverse clinical signs just before and immediately after dosing and prior to each blood collection time point, with no such signs noted. Samples were collected from restrained nonsedated animals, immediately transferred to prechilled tubes containing ∼1 mg dipotassium ethylenediaminetetraacetic acid (K_2_EDTA) as anticoagulant and 20 μl of 5 mM dichlorvos solution as stabilizer, mixed, and placed on ice until plasma was prepared by centrifugation (3,000 × *g*, 10 min, 2 to 8°C). Immediately after centrifugation, 200 μl plasma was extracted with 4 volumes chilled MeOH-acetonitrile (ACN), 3:1 (vol/vol), and internal standards. The supernatants were clarified by centrifugation (12,000 rpm, 15 min, 4°C), and aliquots were stored at −60°C until they were analyzed by LC-MS/MS as described below. After the terminal blood collection, animals were anesthetized with an intravenous injection of pentobarbital sodium (60 mg/kg), and duplicate samples (∼1 g) of lung, liver, and kidney tissues were collected from each animal at the same organ location, snap-frozen in liquid nitrogen, and stored at −60°C. The frozen organs were broken into small pieces, and approximately 0.5 g of each tissue was homogenized in 5 volumes (wt/vol) prechilled homogenization solution (30% 268 mM K_2_EDTA adjusted to pH 7.2 to 7.4 with KOH, 70% MeOH, final pH 7.8) with internal standards, in a dry ice-ethanol bath. After centrifugation (13,000 rpm, 10 min, 4°C), the supernatants were stored at −60°C or below until LC-MS/MS analysis. Tissue samples from an untreated cynomolgus monkey were collected, processed, and stored as well.

### LC-MS/MS analysis of AT-511, AT-9010, and metabolites.

Samples were prepared for MS analysis by drying aliquots under nitrogen and reconstituting them in water or 10 mM ammonium bicarbonate buffer (pH 9), vortexing vigorously for 15 min. For AT-9010 in cells and tissue homogenates, 7 μl was injected onto an Ascentis Express C_18_ (100 by 4.6 mm), 2.7-μm high-pressure liquid chromatography (HPLC) column with a Sciex Triple Quad 6500 mass spectrometer (electrospray ionization [ESI] positive ion, multiple reaction monitoring [MRM] mode). A binary nonlinear gradient with mobile phases A (2% acetic acid, pH 8.5) and B (MeOH) was used to elute samples at 0.7 ml/min, with a run time of 5.6 min. For the active TP from ALS-8112, 3-μl samples were injected onto an Agilent Zorbax Extend C_18_ (50 by 2.1 mm) 5-μm column with Sciex API 6500 mass spectrometer in ESI negative ion, MRM mode. A binary nonlinear gradient with mobile phases A (0.001% ammonium hydroxide and 0.18 mM dibutyl acetic acid in water, 1:1 [vol/vol]) and B (MeOH) was used to elute samples at 0.7 ml/min, with a run time of 7 min. To measure AT-511, AT-551, and AT-273 in plasma and tissues, 6-μl samples were injected onto a Gemini C_18_ (50 by 4.6 mm) 5-μm column at 40°C and a Sciex QTRAP 6500 mass spectrometer (ESI positive ion, MRM mode). A binary nonlinear gradient with mobile phases A (0.1% formic acid in water) and B (0.1% formic acid in ACN, 2%, 30%, 98%, and 2%) was used to elute samples at 0.8 ml/min, with a run time of 5 min. Standards in 50% MeOH were used for calibration. Ions monitored were *m/z* 540.1/152.1 (AT-9010), 582.3/200 (AT-511), 464.2/313.1 (AT-551), and 300.1/152.1 (AT-273). Internal standards (ISS) as described previously (8) were used to correct for variations in recovery.

### PK data analysis.

Plasma concentrations of AT-511, AT-551, and AT-273 were subjected to noncompartmental pharmacokinetic analysis using WinNonlin software (version 6.3 or above; Pharsight, Mountain View, CA). The linear/log trapezoidal rule was applied in obtaining the PK parameters.

### Prediction of human lung levels of AT-9010 using population pharmacokinetic analysis and simulation.

In a phase 1a study, 18 HCV-infected subjects received 600 mg AT-527 (553 mg equivalent freebase) per day for 7 days, and intensive plasma sampling was performed after the first and last doses for up to 120 h (7) for the measurement of AT-511 and related metabolites, including AT-273. As depicted in Fig. 1, this nucleoside metabolite can only form via dephosphorylation of its intercellular phosphates, including the active triphosphate metabolite AT-9010, and is therefore regarded as a plasma surrogate for the active triphosphate in the liver.

An exploratory population pharmacokinetic (PPK) analysis was performed for AT-273 using the plasma concentration-time data obtained from the 18 subjects. An extravascular model with first-order input and first-order elimination reparametrized into lag time (*T*_lag_), absorption constant (*K*_a_), volume of central compartment (V1), volume of peripheral compartment (V2), clearance (Cl), and intercompartment clearance (Q) with a proportional error model, as implemented in MonolixSuite 2019R1 (Antony, France), was used. This model adequately described the PK of plasma AT-273. Population estimates were 0.46 (standard error, ±0.00779) h for *T*_lag_, 0.45 (±0.00253) h^−1^ for *K_a_*, 188 (±9.99) liters/h for Cl, 2,070 (±146) liters for V1, 1,270 (±164) liters for V2, and 36.2 (±1.03) liters for Q. These PPK parameters together with the associated variability were then used to simulate plasma PK profiles of AT-273 for various dosing regimens based on a 550-mg tablet using SIMULX as part of MonolixSuite 2019R1. The simulated plasma concentrations of AT-273 were then used to predict lung levels of the active TP metabolite AT-9010 by assuming the plasma AT-273 profiles reflect the liver TP concentrations and then multiplying those concentrations either by the steady-state liver-to-lung AT-9010 concentration ratio (1.6) or the plasma AT-273-to-lung AT-9010 concentration ratio (1.2) observed at 12 h (trough) after the last of 6 BID doses of AT-527 administered orally to NHPs as described above.
